# Trait Emotional Intelligence and English Language Achievement Among University EFL Learners: A Global and Dimensional Analysis

**DOI:** 10.3390/jintelligence14070148

**Published:** 2026-07-14

**Authors:** Aser Altalib

**Affiliations:** Department of English, College of Arts, Jouf University, Sakaka 72341, Saudi Arabia; aaltalib@ju.edu.sa

**Keywords:** trait emotional intelligence, TEIQue-SF, dimensional analysis, English language achievement, individual differences, EFL learners

## Abstract

Trait emotional intelligence (EI) has been consistently associated with academic achievement, yet most empirical work has used global EI scores rather than examining how its four dimensions (well-being, self-control, emotionality, and sociability) contribute differentially. The present study addresses this gap through a dimensional analysis of trait EI as a correlate of English language achievement in a sample of 666 Saudi undergraduate students. Participants completed the Short Form of the Trait Emotional Intelligence Questionnaire (TEIQue-SF), and their final English course grades served as the measure of language achievement. Global trait EI correlated positively with achievement (*r* = 0.32), and the four dimensions jointly accounted for 16.6% of the variance, more than the 10.5% accounted for by the global score alone. Emotionality showed the strongest unique association with achievement (β = 0.35), followed by self-control (β = 0.13), while the well-being coefficient was negative in the unadjusted dimensional model (β = −0.14). After adjustment for gender, age, and academic major, emotionality and self-control remained the only significant EI dimension-level correlates of grade. Overall, the findings support reporting trait EI at both global and dimensional levels, with the strongest interpretation placed on emotionality and self-control.

## 1. Introduction

Over the past two decades, emotional intelligence (EI) has become one of the most actively researched individual-difference constructs in educational psychology. The meta-analytic evidence supports a positive association between EI and academic achievement across educational levels and outcome measures: MacCann et al. (2020), drawing on 158 independent samples, documented a significant overall effect that held across different EI conceptualisations and different academic indicators. However, much of this evidence has been generated at the level of the global EI score rather than at the level of the underlying dimensions. As Petrides (2009) has argued, trait EI is hierarchically organised, comprising four broad dimensions (well-being, self-control, emotionality, and sociability) that aggregate to a global score. Whether these dimensions contribute differentially to academic achievement, or whether the global score is essentially sufficient, remains an empirical question that has received comparatively limited attention in the literature.

Within the broader EI literature, an important distinction is drawn between ability EI, which conceptualises emotional intelligence as a set of cognitive abilities assessed through performance-based tasks (Mayer & Salovey, 1997), and trait EI, which conceptualises emotional intelligence as a constellation of emotion-related self-perceptions assessed through self-report instruments (Petrides & Furnham, 2001). The present study adopts the trait EI framework on the grounds that learners’ beliefs about their own emotional capacities are likely to shape how they engage with the demands of academic tasks (Petrides, 2009; Petrides et al., 2016). Within this framework, the four dimensions are conceptually distinct (well-being, self-control, emotionality, and sociability) but empirically correlated. Whether they carry independent predictive value for academic outcomes, or whether the global score captures most of the variance they share, is an empirical question with implications for how the construct should be used in practice.

Language learning provides a particularly suitable empirical context for testing the dimensional structure of the relationship between trait EI and achievement. Unlike many other academic domains, learning a foreign language requires learners to communicate, make mistakes, and be evaluated in real time, often in front of peers (Dewaele & MacIntyre, 2014; MacIntyre & Gregersen, 2012). The affective demands of this process (anxiety, frustration, and uncertainty during target-language use) have been shown to suppress communicative engagement and reduce classroom participation, with downstream consequences for achievement (Alrabai, 2022; Dewaele et al., 2018). Within Pekrun’s (2006) Control-Value Theory, learners’ appraisals of control and value shape the emotions they experience during learning, and emotion-related dispositions such as trait EI are accordingly treated as a source of variability in learning outcomes (Pekrun, 2024; R. Wang & Xu, 2026). Two recent meta-analyses have established the global link between trait EI and language achievement with reasonable precision. Y. Wang and Liu (2023), synthesising 39 studies, reported a moderate-to-large positive overall effect. Peng and Shuhong (2025), drawing on 47 studies across eight countries, obtained a small-to-moderate correlation (*r* = 0.24 for subjective achievement; *r* = 0.41 for objective achievement; *r* = 0.37 for course grades). Dimensional evidence, however, remains comparatively thin: Chen and Zhang (2020) examined the four trait EI dimensions in a sample of 72 Chinese postgraduate English as a foreign language (EFL) learners, finding that global trait EI correlated significantly with overall English performance and that well-being emerged as the strongest dimension-level predictor of listening. This leaves open whether similar dimensional patterns would appear in larger samples and when language achievement is operationalised through course grades.

Three patterns in this literature converge to motivate the present study. The first and most central is that the differential contributions of the four trait EI dimensions to course-graded language achievement remain underexamined. The closest dimensional precedent, Chen and Zhang (2020), drew on a sample of 72 postgraduate learners and on an institutional proficiency test rather than course grades, and a sample large enough to support stable dimensional estimates against a course-grade outcome has not yet been examined. The second is that the empirical literature has been geographically concentrated in Chinese and Iranian EFL contexts, with limited representation of Arabic-speaking learners; in Peng and Shuhong’s (2025) meta-analysis, only one of the 47 included studies was drawn from a Saudi sample. The third is that, although Saudi research on individual differences in L2 learning has begun to develop (most notably through Altalib’s (2019) large-scale work on L2 motivation and Alamer and Alrabai’s (2024) validation of a language-domain-specific L2-TEI instrument), few studies have examined the standard four-dimensional structure of the TEIQue-SF as a correlate of course-graded English achievement in this population. The present study addresses these gaps through a dimensional analysis of trait emotional intelligence and English language achievement in a large sample of Saudi undergraduate EFL learners. It attends to both the global trait EI score and the four dimensional subscales, and reports both bivariate correlations and dimensional regression results so that the incremental contribution of dimensional decomposition can be assessed directly.

### 1.1. Trait Emotional Intelligence

Emotional intelligence has been conceptualised from different theoretical perspectives, and these perspectives are not equivalent. The most influential distinction, originally proposed by Petrides and Furnham (2001), is between ability EI and trait EI. Ability EI conceptualises emotional intelligence as a set of cognitive abilities related to emotional information processing, measurable through performance-based tasks in a manner similar to cognitive ability tests (Mayer et al., 1999; Mayer & Salovey, 1997). Trait EI, by contrast, is defined by Petrides et al. (2007) as ‘a constellation of emotion-related self-perceptions and dispositions located at the lower levels of personality hierarchies’ (p. 26), and is typically assessed through self-report instruments. In other words, trait EI captures what individuals believe they can do emotionally, rather than what they can demonstrate under controlled testing conditions. The present study adopts the trait EI framework because self-perceptions of emotional competence, rather than objectively measured emotional abilities, are more likely to predict everyday emotional behaviour in contexts such as language classrooms, where learners’ beliefs about their own emotional capacities shape how they respond to challenge and stress (Petrides, 2009; Petrides et al., 2016).

Within this framework, trait EI is commonly described as consisting of four broad dimensions: well-being, self-control, emotionality, and sociability (Petrides, 2009). The well-being dimension reflects individuals’ general sense of optimism, happiness, and self-confidence; self-control refers to the capacity to regulate emotions, manage stress, and resist impulsive behaviour; emotionality involves the ability to perceive and express emotions and maintain close interpersonal relationships; and sociability reflects individuals’ capacity to interact effectively with others and navigate social situations. These four dimensions are not assumed to function in isolation, nor do they typically appear at uniform levels within learners. In this regard, Abu Alkhayr et al. (2022), in a study of Saudi undergraduate students using the TEIQue-SF, found that well-being tends to score highest among the four dimensions, while self-control and emotionality often score relatively lower. This is a pattern that could have implications for understanding which emotional competencies are most developed in this population and which may require greater instructional attention in educational settings.

### 1.2. Trait EI and Language Achievement

A substantial body of research has examined the relationship between EI and academic achievement, and the overall pattern is consistent: emotionally intelligent students may be better able to regulate negative emotions, cope with academic stress, and maintain motivation during demanding learning tasks (Zeidner et al., 2012). The most comprehensive evidence to date comes from MacCann et al.’s (2020) meta-analysis of 158 independent studies, which reported a significant positive relationship between EI and academic performance across educational levels, with the effect holding across different EI measures and different academic outcomes. It should be noted, however, that the strength of this relationship varies. Some studies have reported weaker or non-significant associations, particularly when EI is measured as ability rather than trait, or when achievement is assessed through narrow indicators such as single test scores (MacCann et al., 2020).

Language learning differs from many other academic domains in ways that make EI particularly relevant. It is a particularly public and interactional academic activity, making emotional responses especially salient in classroom contexts. As a result, learners frequently experience anxiety, frustration, and uncertainty when using the target language, and these emotional experiences considerably influence their willingness to communicate, classroom participation, and overall engagement (Dewaele & MacIntyre, 2014; MacIntyre & Gregersen, 2012). Dewaele et al. (2008) demonstrated that learners with higher trait EI reported significantly lower communicative and foreign language anxiety. That is, EI appears to operate as an internal resource that enables learners to regulate the affective demands of language use rather than being overwhelmed by them. Recent empirical work has begun to map the pathways through which EI shapes language outcomes, with studies pointing to anxiety reduction, enjoyment, willingness to communicate, and motivational engagement as central mediators (Dewaele et al., 2018; R. Wang & Xu, 2026).

Empirical research has begun to map the relationship between EI and language achievement more directly. Two recent meta-analyses provide the most comprehensive evidence to date. Y. Wang and Liu (2023), synthesising findings from 39 studies (*N* = 6571) on EI and second/foreign language achievement, found a moderate-to-large positive overall effect that held across a range of educational contexts. Building on and extending this work, Peng and Shuhong (2025) synthesised 47 independent studies from eight countries (63 effect sizes; total *N* = 18,649), distinguishing subjective from objective indicators of language achievement. The pooled correlations they yielded were *r* = 0.24 for subjective achievement, *r* = 0.41 for objective achievement, and *r* = 0.37 specifically for course-grade outcomes, a pattern in which EI was more strongly associated with externally assessed performance than with learners’ own perceptions of their attainment. The strength of the relation between EI and achievement, on their analysis, varied with educational level and the particular language skill assessed, but appeared comparatively stable across different EI instruments and research designs. While meta-analytic evidence for the global link between EI and achievement is therefore well established, dimensional evidence remains thinner. Of particular relevance to the present study, Chen and Zhang (2020) examined trait EI specifically among 72 Chinese postgraduate EFL learners using the TEIQue-SF and the multidimensional framework of Petrides and colleagues. They showed that students’ global trait EI was significantly correlated with their overall English performance (*r* = 0.245, *p* < .01) and significantly predicted their listening and speaking performance, with well-being emerging as the strongest dimension-level predictor of listening; speaking was predicted by self-control, emotionality, and interactions among the dimensions rather than by well-being. While these dimensional differences are suggestive, the modest sample size and the use of an institutional proficiency test rather than course grades leave open the question of whether these dimensional patterns replicate in larger samples and across different operationalisations of language achievement. Partial corroboration comes from Zhang and Zhang (2023), who, drawing on a sample of 181 Chinese first-year English majors, found that all four trait EI dimensions were significantly correlated with L2 willingness to communicate. However, only well-being and sociability emerged as significant dimension-level predictors in regression analyses. The convergence of well-being as a consistent dimensional predictor across these studies suggests that this dimension carries particular importance in language learning contexts, although further evidence is required across different outcome measures and different sociocultural settings.

More recent work has examined the relationship between global trait EI and language achievement within structural-equation frameworks. Xu and Hong (2025), drawing on 801 Chinese ESL learners and using standardised CET-4 scores as the outcome, observed a strong overall effect of global trait EI on language achievement that operated both directly and indirectly through L2 grit and L2 motivation. Their analysis, however, was conducted at the global EI level and used the Wong and Law Emotional Intelligence Scale (WLEIS; Wong & Law, 2002) rather than the TEIQue-SF, leaving open the question of how the four classic trait EI dimensions of the Petrides framework are differentially associated with language achievement.

Two further patterns shape the existing literature. First, the empirical work has been geographically concentrated. The majority of existing studies have been conducted with Chinese and Iranian EFL learners, with comparatively little research from other sociocultural contexts. This pattern is confirmed by Peng and Shuhong’s (2025) meta-analysis, in which only one of the 47 included studies was drawn from a Saudi sample. As Thao et al. (2023) observed in their study of Vietnamese EFL learners, the relationship between EI and language learning outcomes may be shaped by cultural norms regarding emotional expression and academic behaviour, meaning that findings from one context cannot automatically be assumed to transfer to another. Second, although Saudi research on individual differences in language learning has begun to develop, most notably Altalib’s (2019) large-scale study of L2 motivation among 4043 Saudi university students, trait emotional intelligence has received comparatively little attention in this context. The most directly relevant Saudi precedent, Alamer and Alrabai (2024), validated a language-domain-specific L2-TEI instrument and demonstrated its association with language engagement. Their work makes a valuable contribution to Saudi EFL research, but it does not address whether the four classic trait EI dimensions, as captured by the standard TEIQue-SF, are associated with actual English language achievement in this population. In Saudi higher education, where English functions as both an academic and a professional necessity (Elyas & Picard, 2018), this question takes on particular significance.

### 1.3. The Present Study

In sum, the global link between trait EI and language achievement has been well established by the meta-analytic evidence, but the dimensional structure of this link has rarely been examined in a sample large enough to support stable dimensional estimates against a course-grade outcome, and the empirical literature has had little to say about the four trait EI dimensions in Saudi university EFL learners. Drawing on data from a large sample of Saudi undergraduate students taking required university English language courses, the present study addresses the following research questions:

(1) What are the levels of global trait emotional intelligence and its four dimensions among Saudi university EFL students?
(2) Is there a significant relationship between global trait emotional intelligence and English language achievement?
(3) Is there a significant relationship between the four dimensions of trait emotional intelligence (well-being, self-control, emotionality, and sociability) and English language achievement?
(4) To what extent are the four dimensions of trait emotional intelligence uniquely associated with English language achievement?

To address these questions, the analyses examine associations between global and dimensional trait EI scores and course-grade achievement, with the dimensional findings further supported by adjusted models including gender, age, and academic major, sensitivity checks, and model-comparison criteria.

## 2. Materials and Methods

### 2.1. Research Design

The present study employed a quantitative correlational research design to examine the relationship between trait EI and English language achievement among Saudi undergraduate EFL students taking university English language courses. A correlational design was selected because the study aims to examine naturally occurring relationships between students’ trait EI scores and their course grades, rather than manipulate any aspect of their educational experience. This design is consistent with the approach taken by previous studies examining EI in language learning contexts (e.g., Chen & Zhang, 2020; Li, 2020).

### 2.2. Participants and Context

The participants were 666 undergraduate students (*n* = 299 male, 44.9%; *n* = 367 female, 55.1%) enrolled in required English language courses at a Saudi university. Participants ranged in age from 18 to 38 years (*M* = 18.83, *SD* = 1.94). At the time of data collection, all were taking English Language 2, a required course across a range of academic majors, with English Language 1 as its prerequisite. All participants had completed English Language 1 in the previous semester and had received their final grades for that course before completing the questionnaire, so their reported grades reflected actual achieved scores rather than estimates.

The sample was diverse in terms of academic discipline, with participants drawn from four broad academic groupings: medicine and medical sciences (*n* = 294, 44.1%); engineering, computer sciences, and science (*n* = 72, 10.8%); business and administrative sciences (*n* = 146, 21.9%); and social sciences and humanities (*n* = 154, 23.1%). The disciplinary spread is reported to characterise the sample; academic major was also included as a covariate in the adjusted regression models.

Data were collected during the first two weeks of the second semester, before participants had received any feedback on their performance in English Language 2. This timing was chosen to minimise the risk of current course experience influencing participants’ EI self-perceptions. The questionnaire was administered online via Google Forms and distributed to students through their English Language 2 instructors. Because distribution was not handled through a centralised invitation system, the exact number of students who received the invitation was not recorded; a formal response rate therefore could not be calculated. Before completing the questionnaire, participants were informed of the study’s purpose, assured of the confidentiality and anonymity of their responses, and told that participation was voluntary and would not affect their academic assessment. Ethical approval was obtained from the relevant institutional review board prior to data collection, and all participants provided informed consent.

### 2.3. Instrument and Achievement Measure

Trait EI was measured using the Short Form of the Trait Emotional Intelligence Questionnaire (TEIQue-SF; Petrides, 2009), a 30-item self-report instrument derived from the full-length 153-item TEIQue. The TEIQue-SF assesses global trait EI and its four underlying dimensions: well-being (six items), self-control (six items), emotionality (eight items), and sociability (six items), with the remaining four items contributing only to the global score. The instrument has demonstrated strong psychometric properties across multiple languages and cultural contexts (Cooper & Petrides, 2010; Siegling et al., 2015), and TEIQue-SF-based evidence is also available in Saudi higher-education contexts (Abu Alkhayr et al., 2022). Related language-domain-specific trait EI work in Saudi EFL contexts has further highlighted the relevance of emotion-related self-perceptions for language engagement (Alamer & Alrabai, 2024).

For use with Saudi university students, the original English items were translated into Arabic using a forward-backward translation procedure: items were first translated into Arabic by a bilingual translator, independently back-translated by a second bilingual reviewer, and any discrepancies were resolved through discussion. Minor wording adjustments ensured that items were contextually appropriate. A pilot administration with 20 students outside the main sample was conducted prior to main data collection; pilot participants reported no difficulties understanding the items, and internal consistency estimates were satisfactory.

Participants responded to each item on a five-point Likert scale (1 = completely disagree; 5 = completely agree). Higher scores indicate higher levels of perceived emotional competence. The standard TEIQue-SF uses a seven-point response format; the five-point format was selected as an adaptation to the response conventions familiar to Saudi university students, who routinely encounter five-point scales in institutional and educational surveys. The internal-consistency estimates reported in the Results section (α = 0.90 for the global score; α = 0.75 to 0.82 for the four subscales) are consistent with the values reported for the standard seven-point format across cultural contexts (Cooper & Petrides, 2010; Siegling et al., 2015). This indicates that the instrument operated reliably under this adaptation, though the implications for direct comparability with norm data are noted in Section 4.5. Subscale scores were computed by averaging the relevant items for each dimension, and a global trait EI score by averaging across all 30 items, with negatively worded items reverse-scored prior to computation.

In addition to the EI items, the questionnaire included background questions on participants’ gender, academic major, and their final grade in English Language 1, which was reported on the university’s 100-point course-grade scale and served as the measure of second language achievement. The use of course grades as the achievement measure was supported by meta-analytic evidence: both Y. Wang and Liu (2023) and Peng and Shuhong (2025) reported significant associations between EI and achievement for course-grade outcomes across diverse educational contexts, indicating that grades capture learners’ cumulative performance under the cognitive and affective demands of sustained language study. English Language 1 grades also carry substantial academic weight in the Saudi university system, providing a contextually relevant measure of language achievement in formal university English courses.

English language achievement was operationalised as the final course grade in English Language 1, a foundational English course required of all participants prior to enrolment in English Language 2. Grades were awarded on the university’s 100-point course-grade scale, with 60 as the official passing threshold. Because participants were recruited while enrolled in English Language 2, all had already completed and passed English Language 1; consequently, the observed grade range in the analytic sample was 60–100. Participants self-reported their final English Language 1 grade after the grade had been officially issued. Grades were reported as whole numbers on the 100-point scale; the sample mean of 77.58 reported in Section 3.1 is the computed mean of these whole-number grades rather than a feature of how grades were reported.

### 2.4. Data Analysis

The main statistical analyses were conducted using IBM SPSS Statistics (Version 27). The confirmatory factor analysis was conducted in R (R Core Team, 2026) using the lavaan package (Rosseel, 2012; Rosseel et al., 2025). Prior to the main analyses, the dataset was screened for missing values, outliers, and violations of statistical assumptions. The questionnaire was administered online using Google Forms, with responses required for the items used in the present study before submission. As a result, the final analytic dataset contained complete data for the variables included in the reported analyses, and no missing-data imputation procedures were applied. Outliers were examined using standardised residuals and Mahalanobis distance statistics, and no cases warranted removal. The assumptions underlying the main statistical procedures (normality, linearity, homoscedasticity, and the absence of multicollinearity) were checked and no serious violations were noted. Given the large sample size (*N* = 666), the use of parametric procedures was considered appropriate (Tabachnick & Fidell, 2013).

The internal consistency of the TEIQue-SF and its four subscales was assessed using Cronbach’s alpha. Values around 0.70 or above were considered acceptable (Nunnally, 1978); lower coefficients for the shorter subscales were interpreted cautiously in light of the well-documented sensitivity of alpha to scale length (Cooper & Petrides, 2010).

Descriptive statistics (means, standard deviations, minimum and maximum values, skewness, and kurtosis) were calculated for global trait EI, the four EI dimensions, and English language achievement to address Research Question 1.

To address Research Questions 2 and 3, Pearson product-moment correlations were computed between students’ global trait EI score, the four EI dimension scores, and their English Language 1 grade. Effect sizes were interpreted using Cohen’s (1988) general guidelines (*r* = 0.10 small, *r* = 0.30 medium, *r* = 0.50 large), with reference to the field-specific benchmarks recommended by Plonsky and Oswald (2014) for L2 research. Following current recommendations (Cumming, 2014), the results were interpreted primarily through effect sizes and 95% confidence intervals rather than *p* values alone.

To address Research Question 4, a multiple regression analysis was conducted with English language achievement as the outcome variable and the four trait EI dimensions entered simultaneously as predictors, allowing the unique association of each dimension with achievement to be estimated after partialling out the variance shared with the other three. The dimensions were entered simultaneously rather than in nested blocks because the global trait EI score is mathematically derived from the four dimensional subscales and therefore does not constitute a statistically independent predictor; the relationship between global trait EI and achievement is addressed through Research Question 2. For the regression model, *R*^2^, adjusted *R*^2^, *F*, standardised beta coefficients (β), unstandardised coefficients (*B*), standard errors, *t* values, *p* values, 95% confidence intervals, and Cohen’s *f*^2^ were reported. Multicollinearity was assessed using tolerance and Variance Inflation Factor (VIF) values, with VIF > 10 adopted as the criterion for problematic collinearity. All analyses used a significance level of 0.05.

To examine the robustness of the dimensional findings and to address model complexity, several additional checks were conducted. First, a confirmatory factor analysis of the prespecified four-factor structure was conducted with the WLSMV estimator, appropriate for ordinal item-level questionnaire data. Second, the global trait EI model and the dimensional regression model were repeated with gender, age, and academic major included as covariates. For the adjusted models, gender was coded with male as the reference category, and academic major was dummy-coded with Medicine and Medical Sciences as the reference group. Third, four sensitivity regressions were conducted, each omitting one trait EI dimension in turn, to examine the stability of the dimensional coefficients, particularly the coefficient for well-being. Fourth, the Akaike information criterion (AIC) and Bayesian information criterion (BIC) were computed from the exact residual sums of squares for the global and dimensional models, allowing model comparison with an explicit penalty for the number of estimated parameters. Semipartial correlations from the SPSS regression output were also reported as supplementary indicators of each dimension’s unique contribution to explained variance.

## 3. Results

### 3.1. Preliminary Analyses and Trait EI Profile

The internal consistency of the TEIQue-SF and its four dimensions was examined using Cronbach’s alpha. As shown in Table 1, the global 30-item scale demonstrated excellent internal consistency (α = 0.90), and all four subscales fell within the conventionally acceptable range (α = 0.82 for well-being, 0.77 for emotionality, 0.75 for self-control, 0.76 for sociability). The slightly lower coefficients on the four subscales are consistent with the well-documented sensitivity of Cronbach’s alpha to scale length (Cooper & Petrides, 2010), and all four values exceeded the 0.70 threshold proposed by Nunnally (1978). The instrument therefore appeared to function reliably in the present Saudi EFL context, supporting the use of both the global score and the four dimensional subscales in the analyses that follow.

As an additional check on the measurement structure of the adapted five-point Arabic TEIQue-SF, a confirmatory factor analysis of the prespecified four-factor solution was conducted on the 26 dimensional items. The fit indices did not meet conventional criteria for acceptable fit, χ^2^(293) = 3612.93, CFI = 0.858, TLI = 0.843, RMSEA = 0.131, 90% CI [0.127, 0.134], SRMR = 0.110. Inspection of the standardised loadings showed that most items produced interpretable loadings, although a small number were low, including one emotionality item with a near-zero loading (λ = 0.004). For this reason, the dimensional findings reported below are interpreted cautiously as theoretically derived TEIQue-SF subscale scores rather than as evidence of an independently confirmed four-factor measurement structure in the present sample.

The first research question asked about the levels of global trait emotional intelligence and its four dimensions among Saudi university EFL students. Table 2 presents the descriptive statistics for global trait EI, the four dimensions, and English language achievement.

As shown in Table 2, students reported moderately high levels of global trait EI (*M* = 3.56, *SD* = 0.47), with the mean falling well above the midpoint of the five-point response scale. At the dimension level, well-being yielded the highest mean (*M* = 3.99, *SD* = 0.62), followed by sociability (*M* = 3.67, *SD* = 0.60), emotionality (*M* = 3.51, *SD* = 0.57), and self-control (*M* = 3.33, *SD* = 0.62). Following the TEIQue descriptors (Petrides, 2009), these means indicate that students rated themselves highest on the well-being and sociability dimensions and somewhat lower on emotionality and self-control.

The standard deviations of the global EI score and the four dimensions (0.47, 0.62, 0.62, 0.57, 0.60) and the substantial gap between minimum and maximum scores indicate considerable variability in trait EI across the sample. As Chen and Zhang (2020, p. 7) observed in a similar TEIQue-SF analysis of EFL learners, ‘the means of the four factors did not differ greatly from each other’, which holds for the present sample as well: the spread of the four dimensional means was modest (range = 0.66). Skewness and kurtosis values were within conventionally acceptable ranges, with the partial exception of well-being (kurtosis = 2.07), reflecting mild clustering toward the upper end of the scale; given the large sample size (*N* = 666), this departure was not considered problematic for the parametric analyses that followed.

### 3.2. Correlations Between Trait EI and Achievement

The second and third research questions examined the bivariate relationships between trait EI and English language achievement, at the global level and at the level of the four dimensions. Table 3 presents the full Pearson correlation matrix. The correlations among the four dimensions ranged from *r* = 0.50 to *r* = 0.56, and each dimension correlated strongly with the global EI score (*r* = 0.77 to 0.82), indicating that the four dimensions shared substantial variance as facets of the same higher-order construct while remaining sufficiently distinct to warrant separate examination. The correlation matrix is consistent with treating the four dimensions as related facets of a broader trait EI construct while also supporting the analytical decision to examine them individually rather than relying solely on the global score.

Addressing the second research question, students’ global trait EI score was significantly and positively correlated with their English language achievement, *r*(664) = 0.32, 95% *CI* [0.25, 0.39], *p* < .001, a moderate association in Cohen’s (1988) terms, accounting for approximately 10.5% of the variance in achievement (*r*^2^ = 0.105). The magnitude of this correlation is broadly consistent with the pooled effect size for course-grade outcomes reported by Peng and Shuhong (2025) (*r* = 0.37), suggesting that the relationship between global trait EI and achievement in the present Saudi sample aligns with the wider cross-cultural meta-analytic evidence.

Addressing the third research question, all four trait EI dimensions were significantly and positively correlated with English language achievement, although the strength of these relationships differed considerably across dimensions. Emotionality showed the strongest correlation, *r*(664) = 0.38, *p* < .001, a moderate effect, followed by self-control, *r*(664) = 0.28, *p* < .001, and sociability, *r*(664) = 0.25, *p* < .001, both in the small-to-moderate range. Well-being showed the weakest correlation with achievement, *r*(664) = 0.15, *p* < .001, although the effect remained statistically significant. In a supporting adjusted model, global trait EI remained associated with English Language 1 grade after adjusting for gender, age, and academic major, *B* = 7.32, β = 0.33, *p* < .001.

### 3.3. Regression Analysis

The fourth research question asked to what extent the four trait EI dimensions are uniquely associated with English language achievement. A multiple regression analysis was conducted with English Language 1 grade as the outcome variable and the four trait EI dimensions entered simultaneously as predictors. Table 4 presents the regression coefficients, confidence intervals, and collinearity diagnostics.

The overall model was statistically significant, *F*(4, 661) = 32.78, *p* < .001, accounting for 16.6% of the variance in English language achievement (*R*^2^ = 0.17, adjusted *R*^2^ = 0.16), with Cohen’s *f*^2^ = 0.20 corresponding to a medium overall effect (Cohen, 1988). The four trait EI dimensions explained considerably more variance than the global trait EI score alone (10.5%, reported above), suggesting that examining the four dimensions jointly captured aspects of the relationship between trait EI and achievement that the aggregate global score did not. Multicollinearity diagnostics were well within conventional thresholds (all VIF values below 2, range = 1.61–1.75; all tolerance values above 0.57), indicating that the four dimensions could be treated as statistically distinct predictors despite their conceptual relatedness.

At the dimension level, three of the four dimensions showed statistically significant unique associations with achievement after the variance shared with the other dimensions had been partialled out. Emotionality showed the strongest unique association with achievement (*B* = 6.43, *SE* = 0.86, β = 0.35, *t* = 7.50, *p* < .001, 95% *CI* [4.75, 8.12]), followed by self-control (*B* = 2.22, *SE* = 0.77, β = 0.13, *t* = 2.89, *p* = .004, 95% *CI* [0.71, 3.72]). Sociability did not show a significant unique association with achievement (*B* = 1.11, *SE* = 0.82, β = 0.06, *t* = 1.34, *p* = .180, 95% *CI* [−0.51, 2.72]), suggesting that its bivariate association with achievement (reported above) operates largely through variance shared with the other three dimensions.

The semipartial correlations in the unadjusted dimensional model were largest in absolute terms for emotionality (part *r* = 0.27), followed by well-being (part *r* = −0.11), self-control (part *r* = 0.10), and sociability (part *r* = 0.05), indicating that emotionality accounted for the largest unique share of grade variance among the four dimensions.

To assess whether the dimensional pattern held after accounting for demographic characteristics and academic major, the regression was repeated with gender, age, and academic major included as covariates alongside the four trait EI dimensions. The adjusted model accounted for 24.3% of the variance in English Language 1 grades, *R*^2^ = 0.24, adjusted *R*^2^ = 0.23, *F*(9, 656) = 23.45, *p* < .001. Emotionality remained the strongest dimension-level correlate, *B* = 6.35, β = 0.35, *p* < .001, and self-control also remained positively associated with grade, *B* = 2.67, β = 0.16, *p* < .001. The adjusted coefficients for well-being, β = −0.05, *p* = .261, and sociability, β = −0.03, *p* = .572, were small and non-significant. Multicollinearity diagnostics for this model remained within conventional thresholds, with VIF values ranging from 1.18 to 1.93. The corresponding semipartial correlations were 0.26 for emotionality, 0.12 for self-control, −0.04 for well-being, and −0.02 for sociability.

To examine the stability of the dimensional coefficients, four sensitivity regressions were conducted with the unadjusted model, each omitting one trait EI dimension in turn. The key sensitivity pattern concerned well-being: in the full unadjusted model, well-being showed a negative unique coefficient, β = −0.14, *p* = .003, but when emotionality was omitted from the model, the well-being coefficient became smaller and non-significant, β = −0.05, *p* = .248. This pattern is consistent with the view that the negative well-being coefficient in the full dimensional model reflects shared variance with emotionality rather than a simple negative association between well-being and English language achievement.

As an explicit model-complexity check, the Akaike information criterion (AIC) and Bayesian information criterion (BIC) were computed from the exact residual sums of squares of the global trait EI model and the four-dimensional model. Because lower AIC and BIC values indicate better fit after penalising model complexity, both criteria favoured the dimensional model in the unadjusted comparison, ΔAIC = 40.81 and ΔBIC = 27.31, and in the adjusted comparison including gender, age, and academic major, ΔAIC = 36.71 and ΔBIC = 23.20. This supports reporting the dimensional results alongside the global score, rather than treating the dimensional model as simply a more parameter-rich alternative.

Well-being showed a notable sign reversal between the bivariate correlation and the unadjusted dimensional regression model. Although well-being was positively correlated with achievement at the bivariate level (*r* = 0.15, *p* < .001), its unique coefficient was significant and negative in the unadjusted dimensional model (*B* = −2.33, *SE* = 0.79, β = −0.14, *t* = −2.95, *p* = .003, 95% *CI* [−3.89, −0.78]).

## 4. Discussion

### 4.1. Trait EI Profile of Saudi EFL Learners

Taking question one into consideration, the present findings indicate that Saudi undergraduate EFL students report moderately high levels of global trait emotional intelligence, with the four dimensions arranged in a profile in which well-being and sociability sit comparatively higher than emotionality and self-control. Put differently, students appear comparatively more confident in their optimism and social functioning than in their capacity to regulate impulses and to perceive and articulate their own emotional states.

This dimensional ordering closely mirrors the pattern reported by Abu Alkhayr et al. (2022) in their TEIQue-SF study of Saudi undergraduates. The convergence across two independent Saudi samples implies that the profile observed here reflects a relatively stable feature of the Saudi university population rather than a sample-specific finding. One possible explanation is that the well-being domain captures aspects of emotional self-perception that are well supported by the social and familial context surrounding Saudi undergraduates. By contrast, the comparatively lower self-control mean suggests that students view themselves as less assured in their capacity to regulate emotional impulses and manage stress under pressure.

### 4.2. Global and Dimensional Associations with Achievement

Turning to the second and third research questions, the present analyses showed both that global trait emotional intelligence is significantly associated with English language achievement and that the four trait EI dimensions relate to achievement in different ways. The global trait EI score correlated significantly and positively with English language achievement, *r*(664) = 0.32, *p* < .001, a moderate association in Cohen’s (1988) terms that accounted for approximately 10.5% of the variance in achievement. These findings suggest that learners with stronger overall emotional self-perceptions tend to perform better in formal English language courses, even after accounting for the substantial proportion of variance attributable to cognitive, instructional, and contextual factors that lie outside the scope of trait EI. In other words, the link between learners’ beliefs about their own emotional functioning and their academic performance does appear to operate at the global level, consistent with the broader argument made by Petrides (2009) and Petrides et al. (2016) for the predictive validity of trait EI across diverse life domains, and with recent multivariate evidence from Chinese EFL contexts within the same control-value theoretical framework adopted here (R. Wang & Xu, 2026). The magnitude of the present correlation is broadly consistent with the meta-analytic estimates reported by Peng and Shuhong (2025) and Y. Wang and Liu (2023) for second/foreign language achievement, which would imply that the relationship between global trait EI and achievement holds in an Arabic-speaking, Gulf, EFL setting that has so far received limited attention in the empirical literature, and as a result extends the geographical reach of the evidence base on EI and achievement rather than merely replicating it within a familiar context.

This convergence, however, should be interpreted with caution. The correlational design cannot establish the direction of influence between trait EI and achievement, and the global association does not reveal which specific emotional capacities do the work, a question that is taken up in the dimensional analyses below. It should also be noted that not all studies have reported a positive global association between EI and achievement (e.g., Arslan Degirmenci & Yavuz, 2024), which may indicate that the strength of the relationship is sensitive to instrument selection and sample characteristics.

The dimensional analyses provide one such finer-grained view. At the bivariate level, emotionality emerged as the strongest correlate of English language achievement (*r* = 0.38), followed by self-control (0.28), sociability (0.25), and well-being (0.15) (all *p* < .001). It can be argued that the strongest bivariate associations were observed for the dimensions that capture active emotional engagement with the self and with academic tasks, namely emotionality and self-control, rather than for the dimension that captures general positive affect. One possible explanation is that course-grade outcomes depend less on a learner’s general sense of life optimism and more on the specific capacities to perceive what one is feeling during demanding language tasks, to articulate that feeling clearly enough to act on it, and to regulate impulses such as withdrawal, avoidance, or premature task abandonment when language production becomes difficult (Petrides, 2009). Considered together, the dimensional pattern observed here could mean that emotional functioning matters for course grades in a more fine-grained way than the global EI score alone reveals.

The bivariate ordering observed here, in which emotionality is the strongest dimensional correlate of achievement and well-being the weakest, diverges from Chen and Zhang’s (2020) finding that well-being was the strongest dimension-level correlate of overall language proficiency among 72 Chinese postgraduate EFL learners. Three differences between the two studies may bear on this divergence. First, the present analyses draw on a sample nearly ten times the size of Chen and Zhang’s, which supports more stable estimates of dimensional contributions; the relative ordering of dimensions in a sample of 72 is itself sensitive to sampling variability. Second, the outcome measure differs: Chen and Zhang used an institutional proficiency test, whereas the present study used cumulative course grades. Proficiency tests and course grades may tap different processes, with proficiency tests rewarding the demonstration of language ability at a single point in time, and course grades rewarding sustained academic performance over a term under the affective demands of language learning. Third, the two samples differ in academic level (postgraduate versus undergraduate) and in linguistic and cultural background. These differences may indicate that the dimensional structure of how trait EI relates to achievement is genuinely sensitive to sample size, outcome operationalisation, and educational context, and that the apparent inconsistencies across the dimensional literature reflect substantive variation rather than measurement noise.

### 4.3. Dimensional Associations with English Language Achievement

On the fourth research question, the multiple regression analysis produced three findings worth examining in detail. The first concerns the variance explained by the four dimensions jointly. The dimensional model accounted for 16.6% of the variance in English language achievement, *F*(4, 661) = 32.78, *p* < .001, *f*^2^ = 0.20, considerably more than the 10.5% accounted for by the global trait EI score on its own. It can be argued that this provides empirical support for the hierarchical conceptualisation of trait EI proposed by Petrides (2009): the global score and the four dimensional scores are not interchangeable indices of the same construct, and dimensional analysis can capture aspects of the relationship between EI and achievement that global-score analysis is structurally unable to detect.

The second finding concerns the dimensional pattern rather than the global trait EI score alone. After the variance shared among the four dimensions was taken into account, the clearest pattern involved emotionality and self-control, whereas sociability did not retain a significant unique association. One possible explanation is that emotionality and self-control involve the forms of emotional engagement most directly implicated in formal language courses: perceiving and articulating one’s own emotional states, and regulating impulses and emotional reactions under pressure. These aspects of emotional functioning may therefore be especially relevant to course-grade outcomes. Following Petrides’ (2009) descriptors, learners scoring high on emotionality describe themselves as attuned to and capable of expressing their emotions, whereas learners scoring high on self-control describe themselves as able to regulate impulses, manage stress, and control emotional reactions. Both dimensions thus reflect active emotional engagement rather than general emotional tone, and both appear to do work that the other dimensions cannot. The non-significant unique contribution of sociability could mean that its bivariate association with achievement (*r* = 0.25, reported above) operates largely through variance shared with the other three dimensions rather than through anything that is specific to sociability.

The third finding concerns well-being. Although well-being was positively correlated with achievement at the bivariate level (*r* = 0.15, *p* < .001), its coefficient became negative in the regression model after the shared variance among the four trait EI dimensions was controlled (β = −0.14, *p* = .003). The pattern is consistent with the kind of suppression-like dynamic documented by Paulhus et al. (2004) in personality research, in which a moderately correlated predictor with a weak unique relationship to an outcome can show a reversed coefficient once the variance it shares with stronger predictors is partialled out. It should be noted that this finding does not imply that high well-being is detrimental to language achievement; rather, it identifies what is left of well-being once the achievement-relevant variance has been allocated to the other three dimensions. The collinearity diagnostics reported in the regression analyses above (all VIF values below 2, tolerance values above 0.57) make severe multicollinearity an unlikely explanation for the sign reversal. Together with the sensitivity analyses reported above, this pattern can therefore be interpreted, with appropriate caution, as evidence that the part of well-being that is associated with achievement at the bivariate level overlaps substantially with the corresponding portions of emotionality and self-control, leaving a residual that behaves differently. The cross-sectional design cannot, however, definitively distinguish a true suppression effect from other multivariate dynamics, and replication across independent samples is needed before the interpretation can be treated as established.

### 4.4. Implications

The findings of the present study carry implications for trait emotional intelligence as an individual-differences construct, for the methodological treatment of dimensional models in research on EI and achievement, and for EFL pedagogy. These three areas are addressed in turn.

The first implication is theoretical, and it speaks to a longer-running debate about the analytic value of hierarchical versus dimensional structures in trait EI research. The dimensional analyses presented above could indicate that the relationship between trait EI and language achievement is more informatively examined at the dimensional level rather than at the level of the global construct alone. The point is not that the global score is uninformative. In the present analyses, it accounted for a meaningful 10.5% of the variance in achievement, whereas the dimensional model accounted for 16.6% and also revealed the model-dependent reversal in the well-being coefficient. This is consistent with Petrides’ (2009) hierarchical conceptualisation, in which the global score and the four dimensional scores are not interchangeable indices of the same construct and serve different analytic purposes.

The second implication is methodological. When the four dimensions are treated as separate predictors with their own unique relationships to an outcome, patterns can emerge that the global aggregate, by construction, cannot detect. Dimensional studies would therefore benefit from routinely reporting both bivariate and partialled dimensional analyses, allowing suppression-like patterns and other multivariate dynamics to be detected and interpreted rather than concealed.

The remaining implications are pedagogical. The first concerns the type of emotional functioning that appears to be associated with achievement in this sample. Emotionality showed the strongest unique association with achievement (β = 0.35, *p* < .001). This dimension, defined by Petrides (2009) as the capacity to perceive one’s own and others’ emotions clearly and to express them effectively, may indicate that the capacities most directly relevant to course-grade outcomes are those involving active emotional engagement with academic tasks rather than general positive affect. In the EFL context examined here, this could make attention to learners’ affective vocabulary (i.e., the linguistic resources they have for naming and articulating their own emotional states during language learning), a meaningful component of EFL instruction rather than a peripheral concern. Recent work by Lasekan et al. (2025) operationalises this direction by developing a systematic model for integrating emotional-vocabulary instruction into EFL teacher training, and Resnik and Dewaele (2020) found that learners with higher trait EI contribute to more positively toned L2 classrooms, indicating that the affective competencies identified here matter for the classroom climate as well as for individual achievement. In particular, this could include classroom practices that scaffold learners’ affective vocabulary during tasks where anxiety is likely to become salient, such as oral presentations and speaking assessments. Such practices might involve EFL instructors modelling how to name and describe emotions alongside content-related vocabulary, so that learners can articulate what they are experiencing when these tasks become demanding. This form of scaffolding is consistent with the study’s emphasis on emotionality and self-control as the most stable dimension-level correlates of achievement.

A second pedagogical implication concerns the validation of learners’ emotional reactions to language-learning challenges. The relative weakness of self-control in the present sample (*M* = 3.33, the lowest of the four dimensional means), combined with its statistically significant unique contribution to achievement (β = 0.13, *p* = .004), implies that learners in similar contexts would benefit from explicit instructional support in regulating emotional reactions under task pressure. Li and Xu (2019) showed that a classroom-level emotional intelligence training programme with Chinese EFL learners produced measurable increases in both EI and positively toned classroom emotions, evidence that emotional articulation and regulation are responsive to instructional intervention.

A third pedagogical implication concerns teacher training in the Saudi EFL context. The present findings suggest that teacher-training modules focused on recognising and supporting learners’ emotional reactions to demanding language tasks would offer a productive direction for EFL teacher education. Saudi EFL learners contend with both the linguistic demands of English and the social and institutional pressures associated with its use as a medium of instruction (Alrabai, 2022). Almayez (2026) reports that Saudi undergraduates’ emotional engagement during speaking assessment is shaped by their perceptions of fairness, emotional expressiveness, and the perceived usefulness of such assessment. Recent work on English teachers’ attitudes and challenges in ICT-mediated teaching similarly points to the need for structured teacher preparation as classroom demands change (Mahmood et al., 2025). Teacher training would therefore benefit from attending to the affective demands of assessment as well as those of classroom instruction.

### 4.5. Limitations

The findings of the present study should be interpreted in light of several limitations.

First, two measurement decisions warrant explicit acknowledgement. The TEIQue-SF was administered with a five-point response format rather than the standard seven-point format (see Section 2.3), which may limit direct comparability with TEIQue-SF norm data, although the internal-consistency estimates obtained here (α global = 0.90; subscale α = 0.75–0.82) are consistent with the instrument’s psychometric behaviour across cultural contexts (Cooper & Petrides, 2010; Siegling et al., 2015), and administering both formats to comparable Saudi samples could establish their equivalence empirically. In addition, the achievement measure was based on self-reported English Language 1 grades; although the timing of data collection minimised recall risk (see Section 2.2), self-reported grades remain subject to social desirability and recall bias, and verified grade records obtained directly from university administrative systems would strengthen the inferential basis on which the relationship between trait EI and achievement is estimated.

Second, the cross-sectional design cannot establish the direction of influence between trait EI and language achievement. The findings are consistent with possible pathways from trait EI to achievement, but equally consistent with reverse pathways, in which prior academic success shapes learners’ emotional self-perceptions, or with reciprocal pathways in which the two influence one another over time. Longitudinal designs measuring both constructs at multiple time points across a course of study, following the approach of Qualter et al. (2012), could permit stronger causal claims than cross-sectional data alone can support.

Third, all participants were drawn from a single Saudi university, and the patterns observed here may not transfer directly to other Gulf or Arabic-speaking EFL populations. Even within a single Saudi university, Almayez (2026) found significant moderation of speaking-assessment preferences by discipline, gender, and proficiency, implying that the affective patterns examined here likely vary across these subpopulations. Multi-institutional Saudi samples and comparable studies in other Gulf contexts would test the cross-cultural reach of the findings reported here.

A further limitation is that the well-being sign reversal documented in the regression analyses rests on a single sample. Although the methodological literature on suppression effects supports the substantive plausibility of the finding (Paulhus et al., 2004), the interpretation advanced above cannot be treated as established until it has been replicated. Future studies explicitly testing for sign reversals in models estimating associations between trait EI dimensions and language achievement across diverse samples could either corroborate or revise the interpretation offered here.

Three additional methodological limitations should also be noted. First, the confirmatory factor analysis reported in Section 3.1 did not meet conventional criteria for acceptable fit for the prespecified four-factor solution. Although the dimensional alpha coefficients indicated acceptable internal consistency for each subscale, the dimensional findings are best understood as outcomes from theoretically derived TEIQue-SF subscales rather than from a fully validated four-factor structure in the present sample. Second, instructor and class-section identifiers were not recorded, so the analyses could not account for possible clustering of students within instructional sections. Future studies should include section-level identifiers to examine whether associations between trait EI and achievement vary across instructors or classroom groups. Third, because the exact invitation denominator was not recorded, a formal response rate could not be calculated; the analytic sample should therefore be interpreted as a convenience sample of consenting respondents within the English Language 2 cohort.

## 5. Conclusions

The present study is among the few studies to examine the dimensional structure of trait emotional intelligence as a correlate of English language achievement in a large Saudi university EFL sample. By drawing on a sample large enough to support stable dimensional estimates, the study extends earlier dimensional work into a more diverse and demographically distinct learner population. The findings converge with the recent meta-analytic estimates of Peng and Shuhong (2025) at the global level, while diverging from earlier dimensional findings in informative ways.

Two findings carry the main weight of the study. First, global trait EI was positively associated with English Language 1 achievement. Second, the dimensional analyses showed that emotionality was the clearest dimension-level correlate, with self-control also showing a positive association after adjustment. Together, these findings support interpreting trait EI in relation to English language achievement at both the global and dimensional levels, while placing the strongest interpretation on emotionality and self-control.

Taken together, the type of emotional functioning that matters most for course-grade outcomes appears to involve active perception and regulation of learners’ own emotional states rather than general positive affect. Whether this dimensional pattern generalises across additional Saudi institutions, other Gulf and Arabic-speaking EFL populations, and longitudinal designs remains an open question for future research.

## Figures and Tables

**Table 1:** Internal consistency of the TEIQue-SF and its four dimensions (*N* = 666).

Scale	Number of Items	Cronbach’s α
Global EI	30	0.90
Well-being	6	0.82
Self-control	6	0.75
Emotionality	8	0.77
Sociability	6	0.76

*Note.* α = Cronbach’s alpha. *N* = 666.

**Table 2:** Descriptive statistics for global trait EI, the four dimensions, and English language achievement (*N* = 666).

Variable	M	SD	Min	Max	Skewness	Kurtosis
Global trait EI	3.56	0.47	1.93	4.73	−0.43	0.80
Well-being	3.99	0.62	1.50	5.00	−1.07	2.07
Self-control	3.33	0.62	1.33	5.00	−0.22	0.00
Emotionality	3.51	0.57	1.50	4.75	−0.31	0.08
Sociability	3.67	0.60	1.33	5.00	−0.24	0.26
English Language 1 grade	77.58	10.48	60.00	100.00	0.19	−0.67

*Note.* Trait EI variables are scored on a 1–5 Likert metric (higher = higher trait EI); English Language 1 grade is reported on the university’s 100-point course-grade scale and serves as the operational measure of English language achievement in the present study; the observed analytic range in this sample was 60–100.

**Table 3:** Pearson correlations among global trait EI, the four dimensions, and English language achievement (*N* = 666).

Variable	1	2	3	4	5	6
1. Global trait EI	1.00					
2. Well-being	0.79 **	1.00				
3. Self-control	0.77 **	0.52 **	1.00			
4. Emotionality	0.82 **	0.53 **	0.52 **	1.00		
5. Sociability	0.80 **	0.55 **	0.50 **	0.56 **	1.00	
6. English Language 1 grade	0.32 **	0.15 **	0.28 **	0.38 **	0.25 **	1.00

*Note.* ** *p* < .001 (two-tailed). 95% confidence intervals for correlations with English Language 1 grade: global trait EI [0.25, 0.39]; well-being [0.08, 0.23]; self-control [0.20, 0.34]; emotionality [0.32, 0.45]; sociability [0.18, 0.32].

**Table 4:** Multiple regression of English language achievement on the four trait EI dimensions (*N* = 666).

Predictor	B	SE	β	t	*p*	95% CI	Tolerance	VIF
(Constant)	52.89	2.84		18.61	<.001	[47.32, 58.47]		
Well-being	−2.33	0.79	−0.14	−2.95	.003	[−3.89, −0.78]	0.585	1.71
Self-control	2.22	0.77	0.13	2.89	.004	[0.71, 3.72]	0.620	1.61
Emotionality	6.43	0.86	0.35	7.50	<.001	[4.75, 8.12]	0.572	1.75
Sociability	1.11	0.82	0.06	1.34	.180	[−0.51, 2.72]	0.575	1.74

*Note.* Outcome: English Language 1 grade, reported on the university’s 100-point course-grade scale; observed analytic range = 60–100. Model summary: *R*^2^ = 0.17, adjusted *R*^2^ = 0.16, *F*(4, 661) = 32.78, *p* < .001, *f*^2^ = 0.20. SE of the estimate = 9.61.

## Data Availability

The de-identified data that support the findings of this study are available from the corresponding author upon reasonable request, subject to ethics approval conditions.
